# Validation of a point-of-care capillary lactate measuring device (Lactate Pro 2)

**DOI:** 10.1186/s13049-020-00776-z

**Published:** 2020-08-18

**Authors:** Anette Raa, Geir Arne Sunde, Bjørn Bolann, Reidar Kvåle, Christopher Bjerkvig, Håkon S. Eliassen, Tore Wentzel-Larsen, Jon-Kenneth Heltne

**Affiliations:** 1 University of Bergen, Vognstølen 18 C, 5096 Bergen, Norway; 2 Department of Anaesthesia and Intensive Care, Haukeland University Hospital, Bergen, Norway; 3 Helicopter Emergency Medical Services, Bergen, Norway; 4 Department of Clinical Science, University of Bergen, Bergen, Norway; 5 Department of Medical Biochemistry and Pharmacology, Haukeland University Hospital, Bergen, Norway; 6 Department of Clinical Medicine, University of Bergen, Bergen, Norway; 7 Norwegian Navy Special Operations Commando, Norwegian Armed Forces, Bergen, Norway; 8 Haraldsplass Diaconal Hospital, Bergen, Norway; 9 Centre for Child and Adolescent Mental Health, Eastern and Southern Norway, Oslo, Norway; 10 Norwegian Centre of Violence and Traumatic Stress Studies, Oslo, Norway; 11 Centre for Clinical Research, Haukeland University Hospital, Bergen, Norway

**Keywords:** Lactate, Point-of-care, Shock

## Abstract

**Background:**

The measurement of lactate in emergency medical services has the potential for earlier detection of shock and can be performed with a point-of-care handheld device. Validation of a point-of-care handheld device is required for prehospital implementation.

**Aim:**

The primary aim was to validate the accuracy of Lactate Pro 2 in healthy volunteers and in haemodynamically compromised intensive care patients. The secondary aim was to evaluate which sample site, fingertip or earlobe, is most accurate compared to arterial lactate.

**Methods:**

Arterial, venous and capillary blood samples from fingertips and earlobes were collected from intensive care patients and healthy volunteers. Arterial and venous blood lactate samples were analysed on a stationary hospital blood gas analyser (ABL800 Flex) as the reference device and compared to the Lactate Pro 2. We used the Bland-Altman method to calculate the limits of agreement and used mixed effect models to compare instruments and sample sites. A total of 49 intensive care patients with elevated lactate and 11 healthy volunteers with elevated lactate were included.

**Results:**

There was no significant difference in measured lactate between Lactate Pro 2 and the reference method using arterial blood in either the healthy volunteers or the intensive care patients. Capillary lactate measurement in the fingertip and earlobe of intensive care patients was 47% (95% CI (29 to 68%), *p* < 0.001) and 27% (95% CI (11 to 45%), *p* < 0.001) higher, respectively, than the corresponding arterial blood lactate. In the healthy volunteers, we found that capillary blood lactate in the fingertip was 14% higher than arterial blood lactate (95% CI (4 to 24%), *p* = 0.003) and no significant difference between capillary blood lactate in the earlobe and arterial blood lactate.

**Conclusion:**

Our results showed that the handheld Lactate Pro 2 had good agreement with the reference method using arterial blood in both intensive care patients and healthy volunteers. However, we found that the agreement was poorer using venous blood in both groups. Furthermore, the earlobe may be a better sample site than the fingertip in intensive care patients.

## Background

Circulatory shock is usually divided in categories according to the etiology that causes circulatory failure. The cause of circulatory shock among trauma patients is often hypovolemia due to blood loss, while the etiology of non-traumatic shock may be more complex. Circulatory failure and shock develop as the perfusion and oxygen delivery to the organs and tissues decrease and is insufficient to meet the metabolic demand. When oxygen delivery is below a critical level, shock occurs with the accumulation of oxygen debt. Occult shock is the state of early hypoperfusion causing metabolic acidosis, that may occur in patients prior to detectable changes in vital signs. Due to insufficient oxygen delivery to the tissues, anaerobic metabolism leads to the production of lactate [1, 2].

Circulatory failure following both non-traumatic conditions and trauma is common in the prehospital setting, and the mortality rates in patients presenting with shock in the emergency departments are high. Correct initial assessment and identification of shock followed by early resuscitation is important to improve survival of these patients [3, 4].

Prehospital monitoring of vital signs such as systolic blood pressure (SBP) and heart rate (HR) are main indicators used to identify shock. However, such vital signs often do not change until a patient is near a critical stage, and therefore often fail to predict shock at an early stage [5–7]. Blood lactate monitoring is widely accepted in-hospital as an indirect marker of tissue hypoxia in critically ill patients. The Surviving Sepsis Campaign Guidelines use a cut-off value of greater than 4 mmol/L lactate for early resuscitation therapy to treat sepsis [8]. Lactate levels are commonly measured during resuscitation to predict mortality and to evaluate and guide treatment in the emergency department (ED) and the intensive care unit (ICU). However, lactate levels are infrequently measured in the prehospital setting [9, 10].

Recent prehospital studies suggest that lactate may be more sensitive than SBP and HR in identifying haemorrhagic shock patients who may benefit from early blood transfusion, and suggest that lactate may be independent of conventional vital signs in identifying these patients [10–14]. Implementation of point of care lactate monitors in emergency medical services (EMS) may contribute to the evaluation of patients in the early stages of shock, thereby establishing a trigger for resuscitation [15, 16].

The primary aim of this study was to validate the handheld device Lactate Pro 2 (LP2) in two different groups: healthy volunteers and critically ill patients. The secondary aims were to compare capillary blood lactate in the fingertip and earlobe with arterial blood lactate to investigate whether capillary lactate reflects the actual arterial levels.

## Methods

### Study design

This is a prospective observational study. The study was performed with two groups: haemodynamically compromised intensive care patients and healthy volunteers performing a maximal oxygen consumption test (VO2 max test). The healthy volunteer group had a longitudinal study design with repeated measurements over a short period of time.

### Setting

The study took place in the Intensive Care Unit, Haukeland University Hospital, Norway**.** The healthy volunteers were tested at the VO2 test laboratory at the same hospital. The study was conducted from September 2016 to February 2018.

### Participants

Patients were enrolled from the ICU at Haukeland University Hospital. Inclusion criteria for intensive care patients were (i) adults at least 18 years of age, (ii) an arterial line and a central venous catheter, and (iii) informed consent from the next of kin or the patient him- or herself if awake and competent to give consent. Healthy volunteers were enrolled from the medical faculty in Bergens sports team. Inclusion criteria for the healthy volunteers included were (i) adults at least 18 years of age and (ii) informed consent.

### Sampling

Blood gases from the arterial and central venous lines of the ICU patients were sampled in heparinized syringes by the ICU nurses. They were analysed on both the LP2 and the ABL800 Flex (ABL) immediately. Capillary blood was drawn from the fingertip and earlobe and analysed on the LP2. Two LP2 instruments, denoted LP2.1 and LP2.2, were used for quality assessment: the first drop of blood was measured on the LP2.1 and the second on LP2.2. In one ICU patient only one arterial and one venous sample was drawn. Two capillary samples each from fingertip and earlobe were sampled. All capillary samples were measured by author A.R. The timing of sampling with respect to day of ICU treatment was not registered, but the patients were included shortly after ICU admission. The healthy volunteers underwent a VO2-max test on treadmill (Modified Bruce Protocol) with an arterial line and a peripheral venous catheter during the test [17]. Repeated measurements of arterial, venous and capillary blood lactate in this group were recorded at rest, directly after the VO2-max test and at 3, 5, 10 and 20 min after the test was completed. Capillary blood samples were drawn from the fingertip and earlobe. The arterial and venous blood samples were drawn in heparin tubes, stored on ice and analysed on the ABL within 30 min.

### Materials

Lactate Pro 2 (AKRAY Europe B.V. Prof J.H Bavincklaan 51,183 AT, Amstelveen, the Netherlands) is a handheld point-of care analyser that operates by enzymatic amperometric detection. Blood lactate reacts with the reagent on the test strip, which produces a small electrical current proportional to the concentration of blood lactate. The meter measures this current and calculates the blood lactate level. It requires 0.3 μl of a whole-blood sample and 15 s to measure the lactate value. LP2 has a measurement range between 0.5–25.0 mmol/L. If “Hi” or “Lo” appears on the display it means that the blood lactate level is above 25.0 mmol/L or below 0.5 mmol/L**,** respectively. Therefore, in this study only lactate values between 0.5 and 25 were included.

ABL800 Flex (Radiometer Medical ApS, Åkandevej 21, DK-2700 Brønshøj, Denmark) is the standard blood gas machine used in the ICU at Haukeland University Hospital. It uses an amperometric method to measure the lactate value. The enzyme lactate oxidase converts lactate to H_2_O_2_ and the oxidation of H_2_O_2_ produces an electrical current that is directly related to the amount of lactate. The analyser thereby automatically calculates the lactate concentration in the sample.

### Statistical methods

Logarithmic transformation was performed for lactate since lactate has a skewed distribution and logarithmically transformed lactate is closer to normal distribution. Due to the repeated measurements in each individual in the healthy volunteer group, a mixed effects model, with log transformed lactate as dependent variable, with instrument and time as independent variables was used. Fixed effect coefficients were exponentially transformed to be interpretable as ratios. First, we estimated a model with the same difference between instruments at all time points and then a model when this was not assumed. For ICU patients, mixed effects models were used for the measurements at different sites and with different instruments, and Bland-Altman plots were used to compare different instruments and different sites [18]. Calculations were performed with R (R Foundation for Statistical Computing, Vienna, Austria) using the R package nlme for mixed effects analysis [19].

## Results

Forty-nine ICU patients and 11 healthy volunteers were included. Of the healthy volunteers there were 4 women and 7 men. The mean age was 24.5 years (Table 1). In the ICU patients there were 41 missing values (6.3%), including 34 values above the detection limit. In the healthy volunteers, there were 10 missing values (2.5%), including 4 values above the detection limit.

**Table 1:** Demographic data of ICU patients

Gender	N (%)
**Male**	**28 (57.0%)**
**Female**	**16 (33.0%)**
**NA**	**5 (10.0%)**
**Mean age**	**62.0 ± 17.1 years**
**Diagnosis**	
**Sepsis**	**15 (30.6%)**
**Hypovolemic shock**	**4 (8.2%)**
**Trauma**	**4 (8.2%)**
**Respiratory failure**	**3 (6.1%)**
**Severe burn injury**	**3 (6.1%)**
**Cardiogenic shock**	**4 (8.2%)**
**Other**	**11 (22.4%)**
**NA**	**5 (10.2%)**

### Instrument comparisons

**Fig. 1:**
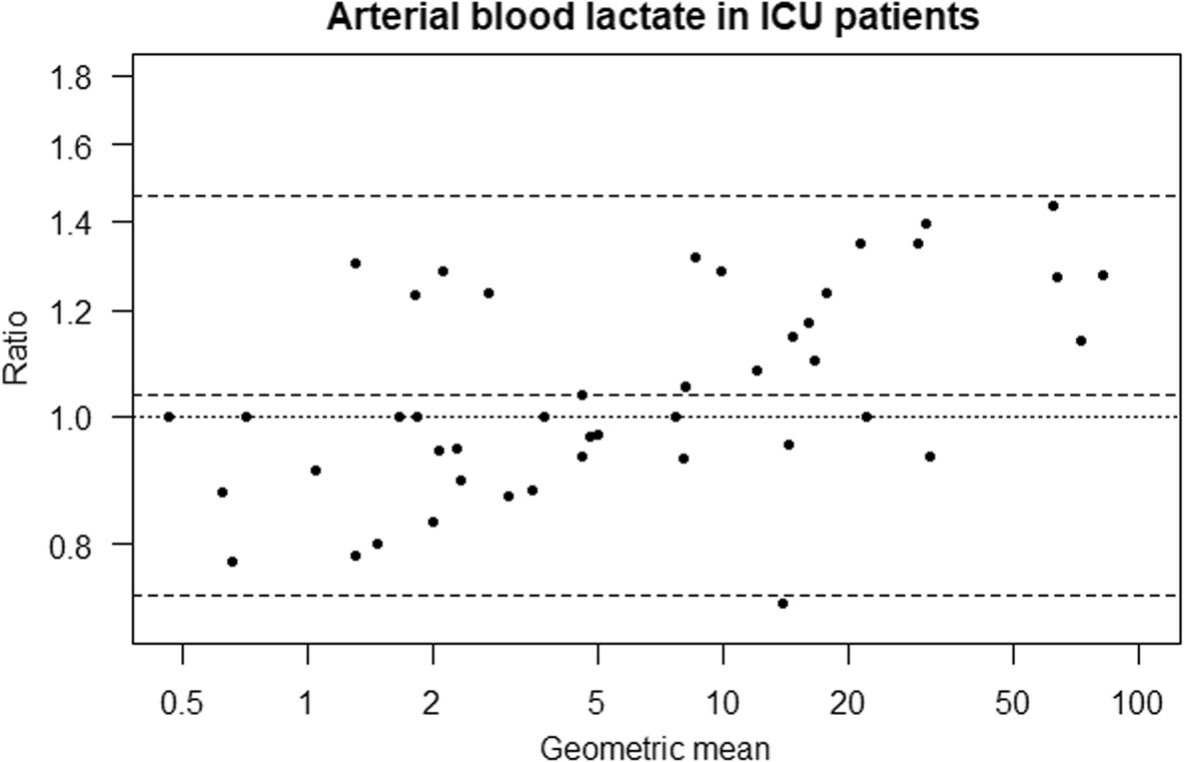
Bland-Altman plot for instrument comparison of LP2 and ABL in arterial blood from ICU patients, based on log transformed lactate

**Fig. 2:**
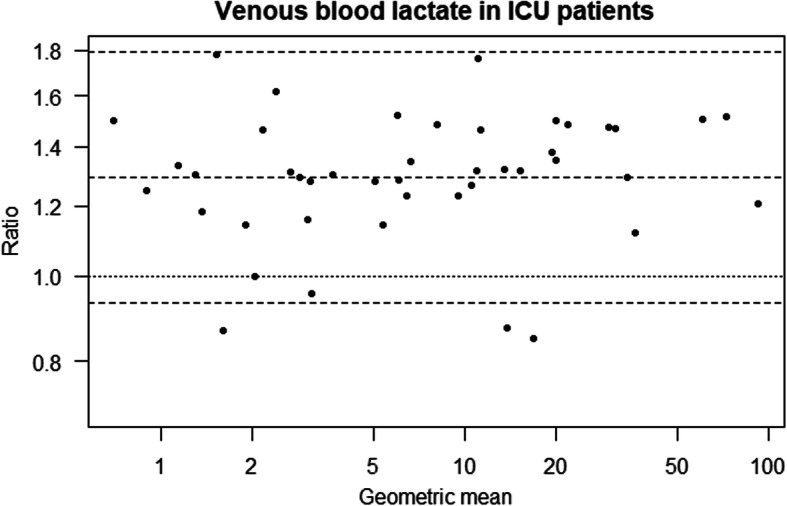
Bland-Altman plot for instrument comparison of LP2 and ABL in venous blood from ICU patients, based on log transformed lactate

In the ICU group (*n* = 49), we found no significant difference in measured lactate between the LP2 and the ABL using arterial blood (Table 2, Fig. 1). We found significantly higher values with LP2 than ABL using central venous blood (Table 2, Fig. 2).

**Table 2:** Results of instrument comparisons with arterial and venous blood in ICU patients and healthy volunteers

	Estimate (Ratio)	95% CI	***p***-value
**ICU patients**			
Arterial			
LP2.1 vs ABL	1.03	0.99 to 1.08	0.140
LP2.2 vs ABL	1.04	0.99 to 1.09	0.102
LP2.2 vs LP2.1	1.004	0.96 to 1.05	0.871
Venous			
LP2.1 vs ABL	1.29	1.24 to 1.35	< 0.001
LP2.2 vs ABL	1.29	1.23 to 1.35	< 0.001
LP2.2 vs LP2.1	0.998	0.96 to 1.04	0.938
**Healthy volunteers**			
Arterial, LP2 vs ABL	0.96	0.93 to 1.002	0.063
Venous, LP2 vs ABL	1.07	1.03 to 1.11	0.001

The results presented in the table are based on mixed effects models with log transformed lactate as the dependent variable. The ratios presented are based on fixed effect coefficients, exponentially transformed to be interpretable as ratios. For example, arterial lactate measurements on the instrument LP2.1 are estimated as 3% higher than measurements on the ABL in ICU patients

In the healthy volunteer group (*n* = 11), we found no significant difference between LP2 and ABL using arterial blood but significantly higher values for LP2 when using peripheral venous blood (Table 2). We found no statistically significant difference between LP2.2 and LP2.1 in both arterial and venous blood. In a mixed effects model where the instrument discrepancy could vary during follow-up, LP2 measured lower values in arterial blood compared to ABL at “rest” (data not shown), but we found no significant difference between LP2 and ABL at the other time points.

### Sample site comparisons

**Fig. 3:**
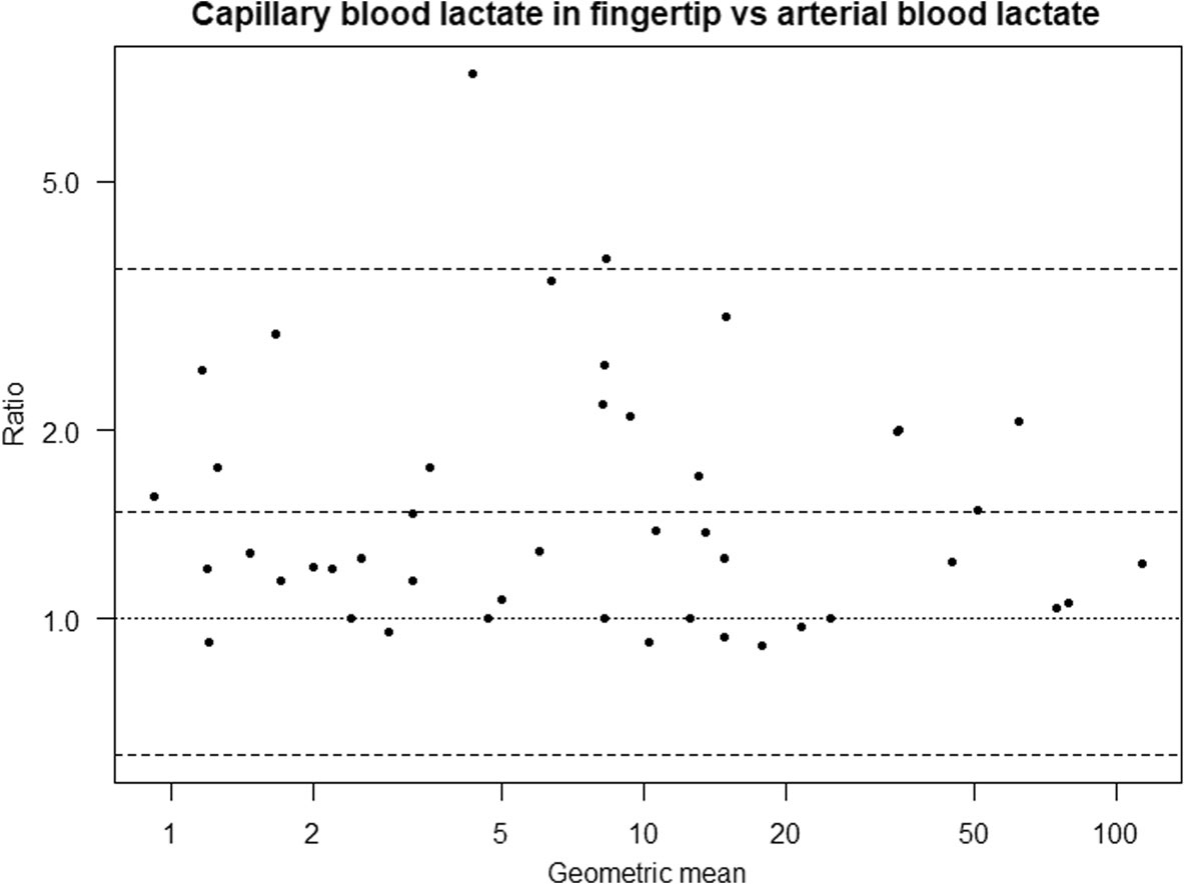
Bland-Altman plots for comparison between lactate in fingertip with arterial lactate in ICU patients, based on log transformed lactate

In the ICU group, we found significant differences in both fingertip and earlobe compared to arterial blood using the LP2.1. Capillary blood lactate in the fingertip and earlobe was 47% (95% CI **(**29 to 68%), *p* < 0.001) and 27% (95% CI **(**11 to 45%), *p* < 0.001) higher than in arterial blood, respectively (Table 3). When comparing fingertip to earlobe we found that capillary blood lactate in the fingertip was 16% higher than in the earlobe (95% CI **(**2 to 32%), *p* = 0.029). Bland Altman plots for comparison between capillary blood lactate in fingertip with arterial blood lactate on both handheld instruments (LP2.1 and LP2.2) in the ICU group are presented in Fig. 3. We observed a rise in the lactate values in the fingertip from the first blood drop measured on LP2.1 to the second blood drop measured on LP2.2. We did not observe the same effect in the earlobe.

**Table 3:** Results of sample site comparisons in ICU patients and healthy volunteers

	Estimate (Ratio)	95% CI	***p***-value
**ICU patients**			
Finger LP2.1 vs Arterial LP2.1	1.47	1.29 to 1.68	< 0.001
Earlobe LP2.1 vs Arterial LP2.1	1.27	1.11 to 1.45	< 0.001
Finger LP2.2 vs Arterial LP2.2	1.85	1.55 to 2.21	< 0.001
Earlobe LP2.2 vs Arterial LP2.2	1.22	1.03 to 1.46	0.024
Finger LP2.1 vs Venous LP2.1	1.14	1.01 to 1.30	0.040
Earlobe LP2.1 vs Venous LP2.1	0.99	0.87 to 1.12	0.871
Finger LP2.2 vs Venous LP2.2	1.43	1.20 to 1.69	< 0.001
Earlobe LP2.2 vs Venous LP2.2	0.95	0.80 to 1.13	0.569
**Healthy volunteers**			
Finger vs arterial	1.14	1.04 to 1.24	0.003
Earlobe vs arterial	0.98	0.90 to 1.06	0.568

The results presented in the table are based on mixed effects models with log transformed lactate as the dependent variable. The ratios presented are based on fixed effect coefficients, exponentially transformed to be interpretable as ratios. For example, lactate measurements from the fingertip are estimated as 47% higher than arterial measurements in ICU patients

In the healthy volunteer group (*n* = 11), we found that capillary blood lactate in the fingertip was 14% higher than arterial blood lactate (95% CI (4 to 24%), *p* = 0.003) (Table 3). We found no significant difference between capillary blood lactate in the earlobe and arterial blood lactate (Table 3).

## Discussion

The LP2 and ABL agreed well in arterial blood taken from both healthy volunteers and intensive care patients. Capillary blood lactate values in the fingertip were significantly higher than the corresponding arterial values in both ICU patients and healthy volunteers. Capillary blood lactate values in the earlobe were significantly higher than arterial blood lactate values in the ICU group but not in the healthy volunteer group.

The earlobe seems to be a better sample site than the fingertip, possibly because it is more central than the fingertip. More importantly, it is less sensitive to variation between two measurements taken consecutively. Contenti et al. used the earlobe as sample site and found that capillary blood lactate was higher than both venous and arterial lactate [20]. In our study, ICU patients had higher capillary blood lactate than arterial blood lactate but almost the same as venous blood lactate.

Studies performed with other handheld devices in ICU populations also report good agreement between capillary and arterial lactate, but these devices were validated in lower lactate ranges [21–23].

Collange et al. is the only other study that compared both capillary and arterial blood samples on a handheld device instead of comparing capillary blood measured on a handheld device with arterial blood measured on a reference method. Like us, they found that capillary lactate was higher than arterial blood lactate. However, in contrast to our findings, they reported that the handheld instrument measured slightly lower lactate values than the reference instrument using arterial blood [22].

Lactate monitoring in EMS may help the provider to detect shock at an earlier stage and may be used as an early trigger for blood transfusion in haemorrhaging patients or for fluid therapy in septic patients [14]. In general, arterial blood is preferred. Capillary lactate should only be measured when rapid measurement is necessary or when arterial blood is not available. Since patients in the prehospital setting in most cases do not receive an arterial line, a handheld capillary blood lactate measuring device may be well suited for EMS services. Measuring lactate values in the field is quick and should not delay other treatments or interventions on-scene. Our results show that LP2 has the potential to overestimate the lactate values, which may lead to overtreatment or over-triage of patients. This may, however, be of less importance than the potential consequences of under-triage in situations where EMS providers fail to detect early stages of shock.

Despite overestimation of the actual value in a single reading, this value may be helpful as an adjunct to other vital signs during assessment of these patients. Further, multiple readings may provide more information because a lactate trend may reflect the clinical course of shock and the effect of resuscitation. This may be valuable in clinical decision making and to guide treatment. When interpreting lactate values, one must consider both the instrumental bias of the LP2 and the physiological discrepancy between arterial and capillary blood lactate values in different sample sites and in different haemodynamical states.

### Strengths and limitations

The strength of this study lies in the standardized procedure for collecting blood and analysing lactate. In contrast to other studies comparing capillary and arterial blood lactate we measured both capillary and arterial/venous blood on the same instrument. Most other studies compare capillary lactate measured on the handheld device with the arterial value measured by the reference method. We believe our method is more correct because it separates the instrument agreement from the difference in the blood samples. We also validated the LP2 in a wide range of lactate values (from 0.5–25.0 mmol/l) in both healthy volunteers and intensive care patients, which shows the differences in lactate distribution in capillary and arterial/venous blood in these two haemodynamically different groups. This study has some limitations of note. We have previously observed that cold LP2 strips may influence the measured value randomly; therefore, we made sure that the LP2 and the strips were stored at room temperature. This limitation in the instrument may have caused a bias in patients with low body temperature. We are considering further research on this issue. The manufacturer states that the device must be used between 5 and 40 degrees Celsius and should be adjusted to the surroundings for at least 20 min. In the pre-hospital environment, the temperature may exceed these limits. This requires the device to be stored in a temperature-controlled environment, e.g. in an isolated casing.

Regarding capillary measurements, the disinfecting agent can cause haemolysis, which may increase the lactate concentration. This study includes few study subjects, which limits the generalizability of the study findings. The number of healthy volunteers included is low due to the ethical aspect of arterial cannulation and the associated risk of complication. The number of missing values in our data set, partly due to the detection limits of the LP2 also constitutes a limitation.

## Conclusion

We found that Lactate Pro 2 had good agreement with the reference method using arterial blood but poorer agreement using venous blood. Our results show the potential for overestimation of the lactate values in haemodynamically compromised patients. The levels of lactate in capillary blood from the fingertip and earlobe were 47 and 27% higher, respectively, than arterial lactate in intensive care patients. The earlobe may be a better sample site than the fingertip in haemodynamically compromised patients.

## Supplementary information

**Additional file 1: Supplementary Table 1.** Healthy Volunteers. **Supplementary Table 2.** ICU patients.

## Data Availability

Data are available upon reasonable request.

## Abbreviations

SBP: Systolic blood pressure
HR: Heart rate
ED: Emergency Department
ICU: Intensive Care Unit
EMS: Emergency medical services
LP2: Lactate Pro 2
VO2: Maximal oxygen consumption test
ABL: ABL800 Flex
CI: Confidence interval

## References

[1] Barbee RW, Reynolds PS, Ward KR (2010). Assessing shock resuscitation strategies by oxygen debt repayment. Shock..

[2] Hooper TJ, De Pasquale M, Strandenes G, Sunde G, Ward KR (2014). Challenges and possibilities in forward resuscitation. Shock..

[3] Holler JG, Bech CN, Henriksen DP, Mikkelsen S, Pedersen C, Lassen AT (2015). Nontraumatic hypotension and shock in the emergency department and the prehospital setting, prevalence, etiology, and mortality: a systematic review. PLoS One.

[4] Vincent JL, De Backer D (2014). Circulatory shock. N Engl J Med.

[5] Andersen LW, Mackenhauer J, Roberts JC, Berg KM, Cocchi MN, Donnino MW (2013). Etiology and therapeutic approach to elevated lactate levels. Mayo Clin Proc.

[6] Jansen TC, van Bommel J, Mulder PG, Rommes JH, Schieveld SJ, Bakker J (2008). The prognostic value of blood lactate levels relative to that of vital signs in the pre-hospital setting: a pilot study. Crit Care.

[7] Rixen D, Siegel JH (2005). Bench-to-bedside review: oxygen debt and its metabolic correlates as quantifiers of the severity of hemorrhagic and post-traumatic shock. Crit Care.

[8] Casserly B, Phillips GS, Schorr C, Dellinger RP, Townsend SR, Osborn TM (2015). Lactate measurements in sepsis-induced tissue hypoperfusion: results from the surviving sepsis campaign database. Crit Care Med.

[9] Dunham CM, Siegel JH, Weireter L, Fabian M, Goodarzi S, Guadalupi P (1991). Oxygen debt and metabolic acidemia as quantitative predictors of mortality and the severity of the ischemic insult in hemorrhagic shock. Crit Care Med.

[10] Bakker J, Jansen TC (2007). Don't take vitals, take a lactate. Intensive Care Med.

[11] Green JP, Berger T, Garg N, Shapiro NI (2011). Serum lactate is a better predictor of short-term mortality when stratified by C-reactive protein in adult emergency department patients hospitalized for a suspected infection. Ann Emerg Med.

[12] Okello M, Makobore P, Wangoda R, Upoki A, Galukande M (2014). Serum lactate as a predictor of early outcomes among trauma patients in Uganda. Int J Emerg Med.

[13] Guyette F, Suffoletto B, Castillo JL, Quintero J, Callaway C, Puyana JC (2011). Prehospital serum lactate as a predictor of outcomes in trauma patients: a retrospective observational study. J Trauma.

[14] Guyette FX, Meier EN, Newgard C, McKnight B, Daya M, Bulger EM (2015). A comparison of prehospital lactate and systolic blood pressure for predicting the need for resuscitative care in trauma transported by ground. J Trauma Acute Care Surg.

[15] van Beest PA, Mulder PJ, Oetomo SB, van den Broek B, Kuiper MA, Spronk PE (2009). Measurement of lactate in a prehospital setting is related to outcome. Eur J Emerg Med.

[16] Shapiro NI, Fisher C, Donnino M, Cataldo L, Tang A, Trzeciak S (2010). The feasibility and accuracy of point-of-care lactate measurement in emergency department patients with suspected infection. J Emerg Med.

[17] Luong MWIM, Taylor CM (2016). Stress testing: a contribution from Dr. Robert a. Bruce, father of exercise physiology. B C Med J.

[18] Bland JM, Altman DG (1999). Measuring agreement in method comparison studies. Stat Methods Med Res.

[19] Team RC (2014). R: A language and environment for statistical computing.

[20] Contenti J, Corraze H, Lemoel F, Levraut J (2015). Effectiveness of arterial, venous, and capillary blood lactate as a sepsis triage tool in ED patients. Am J Emerg Med.

[21] Sabat J, Gould S, Gillego E, Hariprashad A, Wiest C, Almonte S (2016). The use of finger-stick blood to assess lactate in critically ill surgical patients. Ann Med Surg.

[22] Collange O, Garcia V, Kindo M, Meyer N, Lavaux T, Mertes PM (2017). Comparison of capillary and arterial lactate levels in patients with shock. Anaesth Crit Care Pain Med.

[23] Pattharanitima P, Tongyoo S, Ratanarat R, Wilachone W, Poompichet A, Permpikul C (2011). Correlation of arterial, central venous and capillary lactate levels in septic shock patients. J Med Assoc Thail.

