# Supplementary material for: Validation of a point-of-care capillary lactate measuring device (Lactate Pro 2)
Source: Scand J Trauma Resusc Emerg Med. 2020 Aug 18;28:83. doi: 10.1186/s13049-020-00776-z (PMC7437027; doi:10.1186/s13049-020-00776-z)
Supplement: Supplementary file 1 — Additional file 1: Supplementary Table 1. Healthy Volunteers. Supplementary Table 2. ICU patients. [file 13049_2020_776_MOESM1_ESM.docx]

**Supplementary Table 1:** Healthy Volunteers

**Supplementary Table 1:** Mean (min, max), std, 1.IQR, 3.IQR.

|  | Finger LP2 | Earlobe LP2 | Arterial LP2 | Venous LP2 | Arterial ABL | Venous ABL |
| --- | --- | --- | --- | --- | --- | --- |
| Rest | 2.56 (1.10-4.30), 0.78, 2.30, 2.80 | 1.17 (0.7-1.6), 0.30, 1.00, 1.45 | 0.87 (0.60-1.20), 0.18, 0.70, 0.95 | 1.46 (1.00-2.10), 0.32, 1.25, 1.60 | 1.15 (0.70-1.80), 0.31, 0.95, 1.30 | 1.43 (1.00-1.90), 0.28, 1.20, 1.60 |
| Max VO2 | 18.6 (11.6-25.0), 4.6, 15.1, 22.1 | 17.3 (10.6-24.4), 5.2, 12.5, 21.9 | 20.41 (18.7-21.9), 1.2, 19.6, 21.4 | 17.31 (9.2-25.0), 4.6, 14.9, 19.3 | 21.1 (17.0-25.0), 2.6, 19.5, 23.0 | 15.0 (11.5-21.0), 3.5, 11.9, 16.0 |
| 3 min | 19.1 (15.1-24.5), 2.9, 17.0, 20.7 | 18.9 (13.0-24.1), 3.5, 17.0, 21.1 | 20.5 (17.1-24.5), 2.5, 18.4, 22.2 | 18.05 (14.4-21.5), 2.6, 16.0, 20.0 | 20.4 (16.0-24.0), 2.6, 19.0, 21.5 | 16.9 (11.8-20.0), 2.4, 16.5, 18.5 |
| 5 min | 19.4 (15.7-24.9), 2.9, 17.4, 20.5 | 19.5 (16.6-24.9), 3.0, 17.1, 21.5 | 20.6 (16.8-24.7), 2.5, 18.6, 22.3 | 17.9 (14.8-21.6), 2.2, 16.2, 19.9 | 20.5 (16.0-23.0), 2.6, 19.0, 22.5 | 17.3 (12.4-21), 2.6, 16.5, 18.5 |
| 10 min | 19.4 (13.4-24.6), 3.5, 17.2, 22.1 | 19.2 (15.4- 23.3), 2.7, 17.1, 21.3 | 19.9 (16.3-23.9), 2.7, 17.4, 21.5 | 18.35 (13.6-23.1), 2.6, 16.5, 19.7 | 19.8 (15.0-24.0), 2.9, 18.0, 21.5 | 17.7 (12.2-22.0), 3.0, 16.5, 20.0 |
| 20 min | 17.5 (11.5-25.0), 4.6, 13.2, 19.8 | 16.3 (11.6- 23.5), 3.6, 13.5, 18.3 | 16.9 (12.4- 23.0) 3.3, 14.3, 29.1 | 16.4 (12.0-21.1), 2.9, 13.8, 18.9 | 16.6 (11.6-21.0), 3.4, 13.6, 19.5 | 15.0 (10.0-20.0), 3.5, 12.4, 17.0 |

**Supplementary Table 2:** ICU patients

| Finger LP2.1 | Finger LP2.2 | Earlobe LP2.1 | Earlobe LP2.2 | Arterial ABL | Arterial LP2.1 | Arterial LP2.2 | Venous ABL | Venous LP2.1 | Venous LP2.2 |
| --- | --- | --- | --- | --- | --- | --- | --- | --- | --- |
| 6.8 (1.2-25.0), 7.5, 1.8, 8.0 | 5.5 (1.4-13.3), 4.7, 1.8, 9.8 | 5.5 (1.6-21.7), 6.4, 1.6, 21.7 | 5.4 (1.5-21.9), 6.4, 1.7, 6.7 | 4.2 (1.0-16.0), 4.6, 1.4, 5.6 | 4.8 (1.0-20.4), 6.03, 1.4, 6.0 | 4.9 (1.0-21.4), 6.3, 1.4, 5.5 | 4.2 (0.9-16.0), 4.5, 1.5, 5.5 | 3.9 (1.6-8.9), 2.8, 1.6, 5.4 | 5.9 (1.5-24.0), 6.9, 1.7, 7.5 |

**Supplementary Table 2:** Mean (min, max), std, 1.IQR, 3.IQR.
